# Surface Phenotype Changes and Increased Response to Oxidative Stress in CD4^+^CD25^high^ T Cells

**DOI:** 10.3390/biomedicines9060616

**Published:** 2021-05-29

**Authors:** Yoshiki Yamamoto, Takaharu Negoro, Rui Tada, Michiaki Narushima, Akane Hoshi, Yoichi Negishi, Yasuko Nakano

**Affiliations:** 1 Department of Paediatrics, Tokyo Metropolitan Ebara Hospital, Tokyo 145-0065, Japan; 2 Department of Pharmacogenomics, School of Pharmacy, Showa University, Tokyo 142-8555, Japan; tanego@pharm.showa-u.ac.jp (T.N.); rubiaceae1122@yahoo.co.jp (A.H.); yasueco.nakano@gmail.com (Y.N.); 3 Department of Drug Delivery and Molecular Biopharmaceutics, School of Pharmacy, Tokyo University of Pharmacy and Life Sciences, Tokyo 192-0392, Japan; rui.tada@gmail.com; 4 Department of Internal Medicine, Showa University Northern Yokohama Hospital, Kanagawa 224-8503, Japan; narushiz@gmail.com

**Keywords:** allergy, CD45 isoform, extracellular signal-regulated kinases 1/2, oxidative stress, regulatory T cell

## Abstract

Conversion of CD4^+^CD25^+^FOXP3^+^ T regulatory cells (T_regs_) from the immature (CD45RA^+^) to mature (CD45RO^+^) phenotype has been shown during development and allergic reactions. The relative frequencies of these T_reg_ phenotypes and their responses to oxidative stress during development and allergic inflammation were analysed in samples from paediatric and adult subjects. The FOXP3^low^CD45RA^+^ population was dominant in early childhood, while the percentage of FOXP3^high^CD45RO^+^ cells began increasing in the first year of life. These phenotypic changes were observed in subjects with and without asthma. Further, there was a significant increase in phosphorylated ERK1/2 (pERK1/2) protein in hydrogen peroxide (H_2_O_2_)-treated CD4^+^CD25^high^ cells in adults with asthma compared with those without asthma. Increased pERK1/2 levels corresponded with increased Ca^2+^ response to T cell receptor stimulation. mRNA expression of peroxiredoxins declined in T_regs_ from adults with asthma. Finally, CD4^+^CD25^high^ cells from paediatric subjects were more sensitive to oxidative stress than those from adults in vitro. The differential T_reg_ sensitivity to oxidative stress observed in children and adults was likely dependent on phenotypic CD45 isoform switching. Increased sensitivity of T_reg_ cells from adults with asthma to H_2_O_2_ resulted from a reduction of peroxiredoxin-2, -3, -4 and increased pERK1/2 via impaired Ca^2+^ response in these cells.

## 1. Introduction

Regulatory T cells (T_regs_) play critical roles in allergic immune response regulation [1,2,3]. Airway inflammation in paediatric asthma is associated with infiltrating Th2 cells and eosinophils triggered by allergens [4], and most children with asthma are atopic in Japan [5]. Numerous studies have shown that reduced T_reg_ cells [6], decreased levels of forkhead box P3 (FOXP3) protein, a master transcriptional regulator of T_reg_ cells [6,7,8,9], and impaired T_reg_ function result in the loss of control of inflammation [8,10,11,12]. However, several reports have demonstrated increases in the number of T_regs_ during exacerbation of asthma [13,14] and elevated T_reg_ numbers and FOXP3 expression after allergen challenge [15] or glucocorticoid treatment [7,16,17].

Studies have identified that several T_reg_ subtypes, such as thymus-derived T_regs_ (tT_regs_) and peripherally derived T_regs_ (pT_regs_), are involved in the regulation of airway inflammation [18,19,20]. T_reg_ maturation that occurs during development can be detected by a shift in the expression of isoforms of the CD45 surface antigen from CD45RA to CD45RO. This phenotypic switch is a marker of T cell differentiation [21,22,23]: CD45RA^+^ expression is characteristic of naïve T cells before antigen exposure, whereas CD45RO^+^ T cells are memory T cells that have been exposed to antigen. Miyara et al. demonstrated that immature FOXP3^low^CD45RA^+^ T_reg_ cells could differentiate to mature FOXP3^high^CD45RA^−^ T_reg_ cells, which were very similar to CD45RO^+^ T_reg_ cells [23]. The cells with the immature phenotype accounted for a substantial fraction of T_reg_ cells in children, whereas the cells with the mature phenotype were dominant in adults. Whereas numerous studies have assessed T_reg_ subtypes in the cord blood and adult peripheral blood, T_reg_ CD45 isoform phenotypic switching during early childhood development has not been examined in allergic subjects. Thus, here we compared changes during development and those with regard to allergies in early childhood.

Mougiakakos et al. reported that the production of high levels of thioredoxin-1 by T_reg_ cells conferred protection against cell death triggered by hydrogen peroxide (H_2_O_2_)-mediated oxidative stress [24]. H_2_O_2_ induces several cellular responses, including the activation of MAP kinase, chemokine production, and apoptosis [24,25,26]. In this study, tolerance to oxidative stress in T_regs_ was compared in paediatric subjects with and without allergy by the assessment of phosphorylation of extracellular signal-regulated kinase 1/2 (ERK1/2) and p38 mitogen-activated protein kinase (p38 MAPK). Moreover, adults with or without asthma were compared with paediatric subjects. Finally, antioxidant stress-related patterns of gene expression were assessed by microarray profiling of Jurkat cells with doxycyline (Dox)-inducible FOXP3.

## 2. Materials and Methods

### 2.1. Subjects

Paediatric subjects, 11 with and 38 without allergy, were recruited at the Showa University Hospital and Tokyo Metropolitan Ebara Hospital between July and December 2004 and between December 2006 and January 2007. Characteristics of all the paediatric subjects are shown in Table 1. The age of paediatric subjects with allergy ranged from 11 months to 13 years and 3 months, and the average age was 4 years and 8 months. The age of paediatric subjects without allergy ranged from 3 months to 8 years and 2 months, and the average age of this group was comparable to that of the allergic paediatric cases. Allergic diseases mainly consist of asthma, allergic rhinitis, atopic dermatitis, and food allergy. The majority of allergic subjects had two or more allergic diseases, and eight of these had simultaneous food allergy and atopic dermatitis. Adult subjects, 10 with and 6 without asthma, were recruited at Showa University Hospital and Showa University Fujigaoka Hospital between July and October 2004 (Table 1). Asthma was defined according to the criteria established by the 1998 Japanese guidelines, the Japanese Pediatric Guidelines for the Treatment and Management of Asthma 2004, and the Global Initiative for Asthma as revised in 2002. Atopic dermatitis and food allergy were defined according to the criteria established by the Japanese Guideline for Atopic Dermatitis 2004 and the Japanese Guideline for Diagnosis and Management of Food Allergy 2005, respectively. All subjects with allergy completed a standard questionnaire regarding allergy symptoms, and atopy was established by the measurement of serum-specific IgE to common allergens such as mite, mold, pet allergens, pollen, and food allergens (CAP RAST system; Pharmacia Diagnostics AB, Uppsala, Sweden). This study was approved by the Showa University Medical Ethics Committee (protocol number 310 for paediatric subjects, protocol number 2002022 for adult subjects) and the Tokyo Metropolitan Ebara Hospital Trust local research and ethics committee. Written informed consent was obtained from all participants or their guardians in accordance with the ethical guidelines of the Declaration of Helsinki: The Materials and Methods should be described with sufficient details to allow others to replicate and build on the published results. Please note that the publication of your manuscript implicates that you must make all materials, data, computer code, and protocols associated with the publication available to readers. Please disclose at the submission stage any restrictions on the availability of materials or information. New methods and protocols should be described in detail while well-established methods can be briefly described and appropriately cited.

Research manuscripts reporting large datasets that are deposited in a publicly available database should specify where the data have been deposited and provide the relevant accession numbers. If the accession numbers have not yet been obtained at the time of submission, please state that they will be provided during review. They must be provided prior to publication.

Interventionary studies involving animals or humans, and other studies that require ethical approval, must list the authority that provided approval and the corresponding ethical approval code.

### 2.2. Flow Cytometry

#### 2.2.1. Cells

Peripheral blood mononuclear cells (PBMCs) were prepared by density gradient centrifugation using Lymphoprep^TM^ (Axis-Shield PoC AS, Oslo, Norway) according to previously reported protocols [11,12]. CD4^+^ T cells were isolated from PBMCs using a MACS CD4^+^ T cell isolation kit II (Miltenyi Biotec, Bergisch Gladbach, Germany) according to the manufacturer’s instructions, and CD4^+^CD25^+^CD127^/low^ T cells were isolated as described previously [12].

#### 2.2.2. Surface Marker Analysis in CD4^+^CD25^+^FOXP3^+^ T Cells

Purified CD4^+^ T cells (1 × 10^6^ cells) were suspended in 1 mL fixation solution (5% paraformaldehyde and 0.5% Tween 20 in phosphate buffered saline [PBS]) and stored overnight at 4 °C. Intracellular FOXP3 staining was performed by anti-FOXP3 monoclonal antibodies (mAbs) conjugated to different fluorochromes, as described previously [26]. To analyse surface antigen expression, staining with Alexa Fluor^®^ 488-FOXP3 mAb was followed by staining for one of the following dual staining paradigms: PE-labelled CD25 (M-A251) plus PC5-labelled CD127, PC5-labelled CD25 (B1.49.9) plus PE-labelled CD45RO (UCHL1), or PC5-labelled CD25 plus PE-labelled CD45RA (HI100). When unlabelled anti-FOXP3 (259D) and biotin-conjugated anti-CD127 (eBiRD5) mAbs (all antibodies were from eBioscience, Inc., San Diego, CA, USA) were used as primary antibodies, cells were incubated with Alexa Fluor^®^ 488- or PC5-streptavidin-labelled secondary antibodies, respectively. The cells were incubated with either non-conjugated or fluorochrome-conjugated antibodies for 15 min at 4 °C. Alexa Fluor^®^ 488-secondary antibody, PE-CD25, PC5-streptavidin, PE-CD45RO, and PE-CD45RA antibodies were obtained from BD Biosciences (Franklin Lakes, NJ, USA) and PC5-CD25 and streptavidin-PC5 antibodies were obtained from Beckman Coulter (Pasadena, CA, USA). The samples were suspended in 350 μL fluorescence-activated cell sorting (FACS) buffer (PBS, 5% fetal bovine serum [FBS], 2 mM EDTA and 0.1% sodium azide) and were analysed by flow cytometry using EPICS XL (Beckman Coulter). The data were analysed by FlowJo software (TreeStar, Ashland, OR, USA). Gating was performed on cells that were highly expressing CD25 (CD25^high^) to increase T_reg_ purity, as the intracellular phosflow labeling was not compatible with FOXP3 co-staining. In the other analyses, CD25 positive cells including both CD25^low^ and CD25^high^ were gated as CD25^+^.

### 2.3. Phosphorylation of ERK1/2 and p38 MAPK

Activation of cellular signaling in CD4^+^CD25^high^ T cells in response to oxidative stress was analysed using BD Phosflow technology (BD Biosciences). Briefly, 3 × 10^5^ PBMCs were suspended in 15 μL of 4-(2-hydroxyethyl)-1-piperazineethanesulfonic acid (HEPES)-buffered saline (107 mM NaCl, 6 mM KCl, 1.2 mM MgSO_4_, 2 mM CaCl_2_, 1.2 mM KH_2_PO_4_, 11.5 mM glucose, and 20 mM HEPES adjusted to pH 7.4 with NaOH) containing 0.1% BSA and incubated with H_2_O_2_ at increasing concentrations from 10 μM to 10 mM for 10 min at 37 °C with agitation at 350 rpm using a reciprocal shaker. After oxidative stress induction, the reaction was stopped by fixing the cells with 250 μL of Cytofix/Cytoperm solution (BD Biosciences) for 10 min at 37 °C. Cells were washed twice with Perm/Wash buffer (BD Biosciences) and resuspended in 50 μL of Perm/Wash buffer. For the analysis of ERK1/2 or p38 MAPK phosphorylation in CD4^+^CD25^high^ T cells, the following fluorescence labelling antibodies were added at 6 μL each and incubated for 60 min at room temperature in the dark: PC5-CD4 (13B8.2; Beckman Coulter), PE-CD25 (M-A251; BD Biosciences), and Alexa488-pERK1/2 (T202/Y204) (612592; BD Biosciences) or Alexa 488-pp38 MAPK (T180/Y182) (612594; BD Biosciences). After washing with the Perm/Wash buffer, the cells were resuspended in 100 μL of FACS buffer and analysed by flow cytometry.

### 2.4. Measurement of Intracellular Ca^2+^ Influx

Changes in intracellular Ca^2+^ influx [Ca^2+^]_i_ were measured using the cell-permeable Fura-2 AM fluorescent dye, as described previously [12,27]. Images of Fura-2-loaded cells were analysed using a video image analyser (Meta Fulora; Nippon Ropper, Tokyo, Japan). Cytosolic Ca^2+^ concentrations were assessed by exciting the cells at 340 and 380 nm using the dual-wavelength excitation method and measuring fluorescence at 510 nm. We have previously shown that Ca^2+^ responses were divided into two categories based on the ratio of peak fluorescence (340/380): poor (<0.1) and robust (>0.1) [12].

### 2.5. Microarray Analysis

RNA was isolated from cells using RNAiso Plus (Takara Bio. Inc., Shiga, Japan). Microarray analysis was performed using the 3D-Gene human oligo chip 25k (TORAY Industries, Tokyo, Japan). RNA sample quality was determined using an Agilent 2100 Bioanalyzer (Agilent Technologies, Santa Clara, CA, USA) and Nanodrop^®^ (Thermo Fisher Scientific, Waltham, MA, USA). RNA amplification and Cy5-labeling were performed using Ambion Amino Allyl aRNA (Thermo Fisher Scientific) and Amersham Cy5 mono-reactive dye (GE Healthcare Bio-Sciences, Pittsburgh, PA, USA), respectively. The Cy5-labeled amplified RNA samples (1 μg per sample) were hybridized at 37 °C for 16 h and scanned using the 3D-Gene Scanner 3000 (TORAY). After subtracting background signal from raw data on each chip, the signal of each gene was normalized by the global normalisation method where the median of the detected signal intensity was adjusted to 25.

Microarray samples were selected and prepared as mentioned below. We have previously generated Jurkat Tet-On^®^ cells with Dox-inducible FOXP3 protein expression: 5G1 cells are control cells, and F2A9 cells are derived from a Jurkat Tet-On^®^ cell line with Dox-inducible FOXP3 [12]. F2A9 cells that were induced with Dox for two days for FOXP3 expression (FOXP3 2-day) demonstrated low [Ca^2+^]_i_ in response to TCR stimulation, similar to that observed in CD4^+^CD25^+^ T cells from subjects without asthma. However, Ca^2+^ responsiveness in F2A9 cells induced with Dox for 5 days (FOXP3 5-day) was similar to that observed in cells from asthmatic subjects [12]. Samples were named Control 2-day, Control 5-day, FOXP3 2-day, and FOXP3 5-day.

Microarray data were analysed for antioxidant gene expression as illustrated by a HeatMap (Supplementary Figure S1). We focused on genes with increased expression in FOXP3 2-day compared with control 2-day, rather than 5-day versus FOXP3 5-day because of the FOXP3 2-day tolerability to oxidative stress. Since FOXP3 5-day is a model cell for asthma T_regs_, the cells are considered more sensitive to oxidative stress than FOXP3 2-day (non-asthma T_regs_). We selected the genes that are able to scavenge reactive oxygen species (ROS) directly for further analyses.

### 2.6. Real-Time Polymerase Chain Reaction

RNA extraction, reverse transcription, and real-time polymerase chain reaction (RT-PCR) were performed as described previously [12]. Peroxiredoxin-2 (Hs99999903_m1) were measured using specific TaqMan^®^ probe primer sets (Life Technologies, Carlsbad, CA, USA), and the mRNA expression of peroxiredoxin-2 (Hs00853603_s1_M), -3 (Hs00428953_g1_M), -4 (Hs01056076_m1_M) and β-actin (NM_005809.5), -3 (NM_014098.3), -4 (NM_006406.1), and β-actin (NM_001101) were quantified using a StepOne^TM^ Real-Time PCR system (Life Technologies). The expression of each gene was normalized to β-actin expression.

### 2.7. Quantification of Glutathione

Glutathione content was measured by the GSH-Glo^TM^ glutathione assay (Promega, Madison, WI, USA) according to the manufacturer’s instructions. Furthermore, 4 × 10^3^ Jurkat cells were suspended in 50 μL of PBS and mixed with 50 μL of 2 × GSH-Glo reagent. Cells were incubated for 30 min at room temperature. After the addition of 100 μL luciferin detection reagent, cells were incubated for 15 min at room temperature. The luminescence was measured at 23 °C with a Tecan Infinite^®^ 200 PRO multimode microplate reader.

### 2.8. Measurement of Caspase Activity

In total, 1 × 10^6^ Jurkat cells were treated with either PBS or 100 μM H_2_O_2_ in 5 mL of RPMI 1640 media containing 0.1% BSA for 4 h in a CO_2_ incubator. The cells were washed twice with PBS, and the supernatants were removed. Caspase activity was measured by the fluorimetric SensoLyte^®^ 520 generic caspase assay (AnaSpec, Fremont, CA, USA) according to the manufacturer’s instructions. Briefly, cells were resuspended in 300 μL of RPMI 1640 media containing 0.1% BSA in a 2 mL tube and incubated with 10 μL of FAM-VAD FMK working solution for 1 h in a CO_2_ incubator. Fluorescence intensity was measured at the excitation wavelength of 490 nm and emission wavelength of 520 nm with Tecan Infinite^®^ 200 PRO multimode microplate reader.

### 2.9. Statistical Analysis

Data were expressed as mean ± standard deviation (SD) unless specified otherwise. Multiple comparisons were calculated by analysis of variance (ANOVA) with a Tukey–Kramer post-hoc test with data comparison among all groups. Data from two groups were analysed by Student’s *t* test. All statistical tests were performed using JMP Pro version 12.0.1 (SAS Institute, Cary, NC, USA).

## 3. Results

### 3.1. Phenotypic Switching of CD4^+^CD25^+^FOXP3^+^ T_reg_ Cells from CD45RA^+^ to CD45RO^+^ during Development in Early Childhood

**Figure 1:**
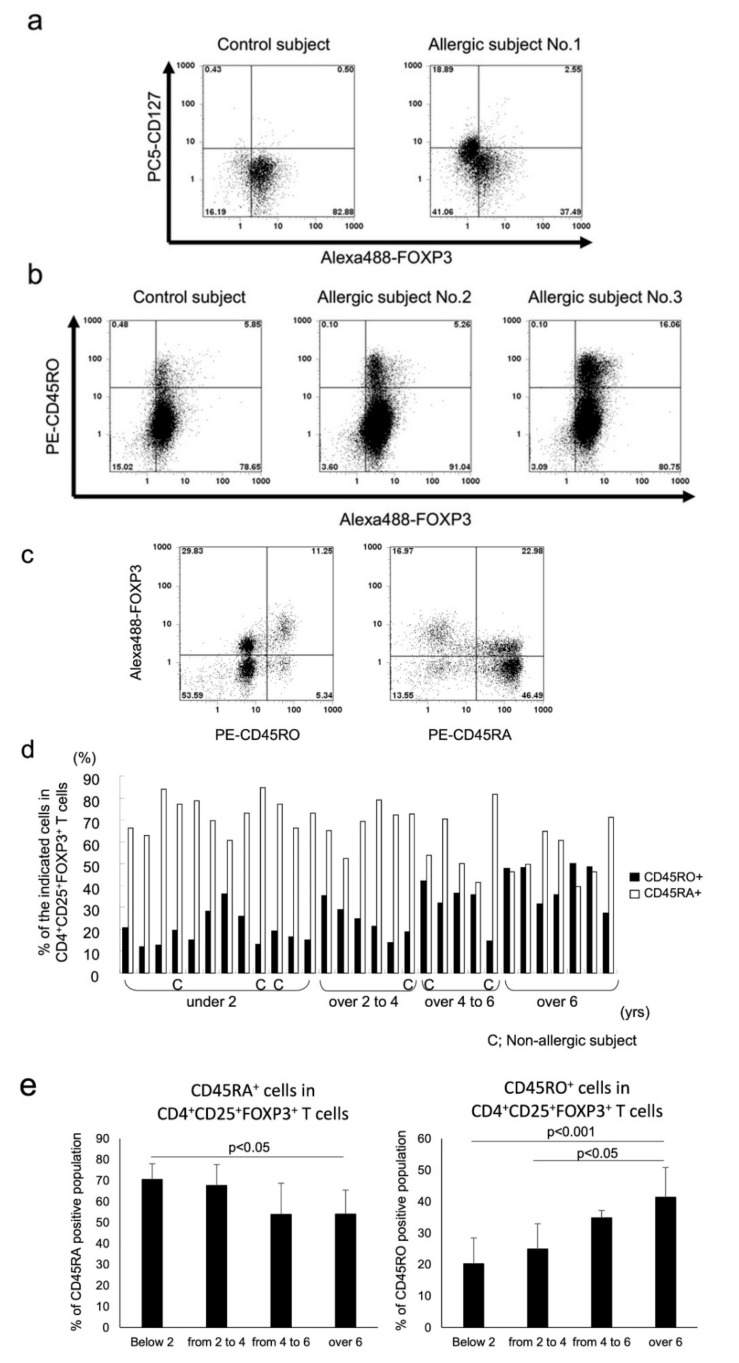
Flow cytometric surface marker analysis of CD4^+^ T cells isolated from paediatric PBMCs are shown from representative cases. In total, 1 × 10^6^ CD4^+^ cells were stained with Alexa 488-FOXP3, PE-CD25, and PC5-CD127 (**a**), or with Alexa 488-FOXP3, PC5-CD25, and PE-CD45RO (**b**). Representative flow histograms are shown. (**c**) The cells were stained with Alexa 488-FOXP3, PC5-CD25, and PE-CD45RO or CD45RA. Representative flow histograms are shown. (**d**) Results from individual paediatric subjects are plotted by bar graph. (**e**) Data from (**d**) were combined into 4 groups by age, and the difference was statistically analysed. All histograms were gated with CD25 because of the analysis of purified CD4^+^ T cells.

The surface marker expression of CD4^+^ T cells in paediatric subjects with or without allergy was determined by flow cytometric analysis. As shown in Figure 1a in a representative flow histogram, the majority of CD4^+^CD25^+^FOXP3^+^ T cells from the control without allergy and subject with allergy (subject with allergy no. 1 in Figure 1a) expressed comparably low or no CD127 (CD127^−/low^). In addition, as shown in representative cases, the percentage of CD4^+^CD25^+^FOXP3^+^ T cells that were CD45RO^+^ was lower in the 1-year-old control and the 1-year-old allergic subject no. 2 that had both food allergy and atopic dermatitis than that in the allergic subject no. 3, an 9.7-year-old child with paediatric allergic asthma (Figure 1b). In agreement with previous studies, FOXP3 expression was much higher in the CD45RO^+^ population than in the CD45RA^+^ population (Figure 1c) [11]. Furthermore, CD45RO expression on CD4^+^CD25^+^FOXP3^+^ T cells was affected by age. Our analysis demonstrated decreases in CD45RA expression with reciprocal increases in the expression of CD45RO beginning at the age of 4 years (Figure 1d,e). However, the percentage of CD45RO population in CD4^+^CD25^+^FOXP3^+^ T cells in the paediatric cohort did not reach the level reported in adults, which is 88.5 ± 7.5% in CD4^+^CD25^high^ T cells and approximately 99% in CD4^+^CD25^very high^ T cells [11,28].

### 3.2. High Tolerance of CD4^+^CD25^high^ T_reg_ Cells from Adults without Asthma to H_2_O_2_-Mediated Oxidative Stress

**Figure 2:**
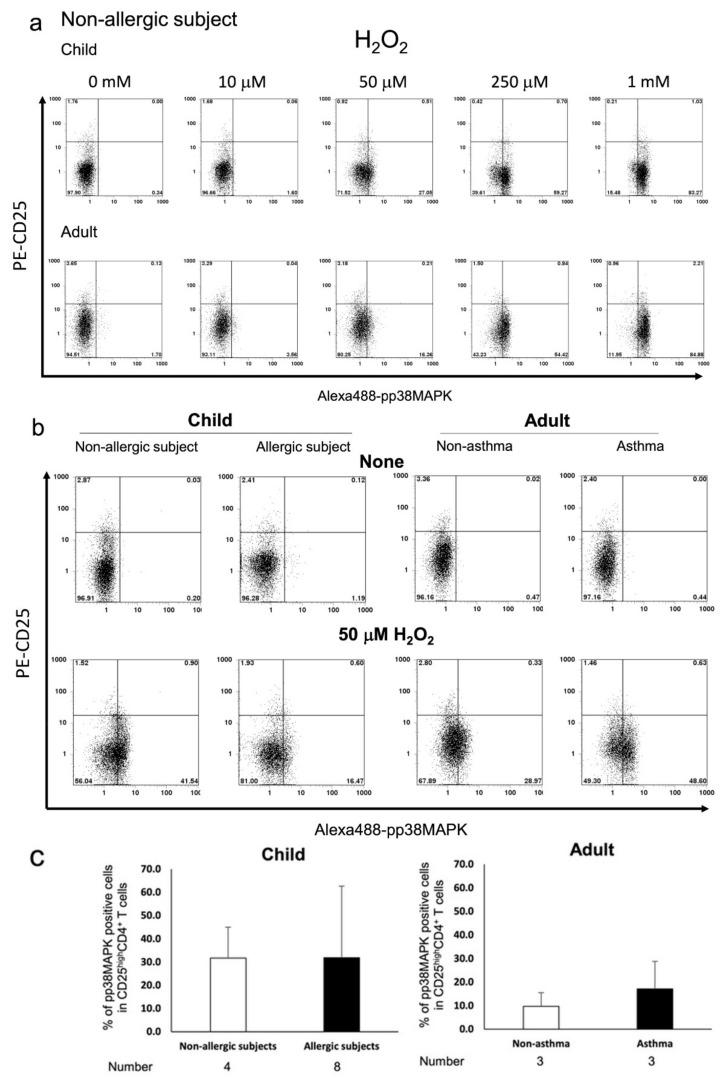
Flow cytometric measurement of p38 MAPK phosphorylation in response to H_2_O_2_ exposure in CD4^+^CD25^high^ T cells from paediatric and adult subjects. 3 × 10^5^ PBMCs were incubated for 10 min and stained with PC5-CD4, PE-CD25, and Alexa 488-pp38 MAPK. Representative flow histograms from paediatric subjects without allergy and adult subjects without asthma are shown (**a**). Phosphorylation of p38 MAPK in cells exposed to 50 μM H_2_O_2_ or vehicle were compared between paediatric subjects with and without allergy and adult subjects with or without asthma. Representative flow histograms (**b**) and average percentage of pp38 MAPK-positive cells are plotted by bar graphs (**c**). Gating was performed on cells highly expressing CD25 antigen.

**Figure 3:**
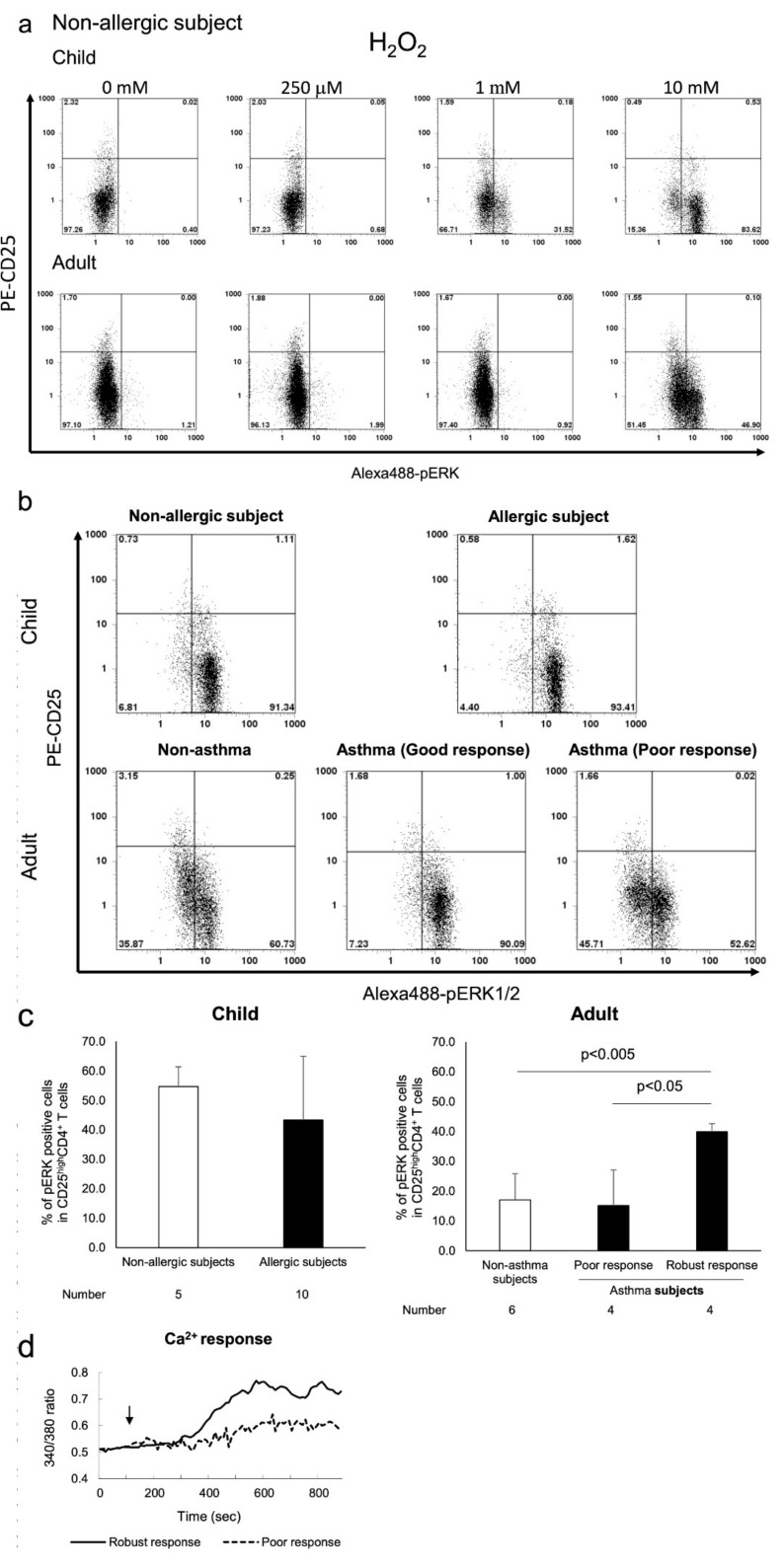
Flow cytometric measurement of ERK1/2 phosphorylation by H_2_O_2_ exposure in CD4^+^CD25^high^ T cells from paediatric subjects without allergy and adult subjects without asthma that were exposed to 10 mM H_2_O_2_ were stained with PC5-CD4, PE-CD25, and Alexa 488-pERK1/2. The representative flow histograms are shown in (**a**). The typical patterns of pERK1/2 expression among all child subject groups are shown in (**b**). The average percentage of pERK1/2- positive cells in all adult subject groups were plotted by bar graphs, and asthma subjects were further divided into two groups by Ca^2+^ response (**c**). Typical patterns (poor response and robust response) of Ca^2+^ response are depicted in (**d**). Gating was performed on cells highly expressing CD25 antigen.

Activation of ERK1/2 signalling by its phosphorylation (pERK1/2) in T_reg_ cells has been shown to be insensitive to T-cell receptor (TCR) stimulation and oxidative stress [29,30], whereas phosphorylation of p38 MAPK (pp38 MAPK) plays an important role in T cell activation [31,32]. Therefore, we analysed pERK1/2 and pp38 MAPK levels in H_2_O_2_-exposed T_regs_ from subjects with allergy. Our results indicated that H_2_O_2_ resulted in a dose-dependent phosphorylation of p38 MAPK in CD4^+^CD25^high^ T_regs_ from paediatric and adult subjects without allergy (Figure 2a). Further, T_regs_ from asthmatic adult subjects were more susceptible to p38 MAPK phosphorylation upon exposure to 50 μM H_2_O_2_ than those from subjects without asthma. The responses of CD4^+^CD25^high^ T_reg_ cells from paediatric subjects with or without allergy to H_2_O_2_ were similar to those observed in the same cell population from adult subjects with allergy (Figure 2b,c). In contrast, ERK1/2 phosphorylation in CD4^+^CD25^high^ T cells from adult subjects without asthma was observed only at high dose H_2_O_2_ (10 mM) treatment (Figure 3a). Measurement of [Ca^2+^]_i_ results in these adult subjects showed that pERK1/2 accumulation induced by 10 mM H_2_O_2_ corresponded with increased influx of Ca^2+^, indicating the characteristic anergic response of these T_reg_ cells [12] (Figure 3b–d). CD4^+^CD25^high^ T_reg_ cells from subjects with increased Ca^2+^ response showed increased pERK1/2 expression, whereas cells with poor Ca^2+^ response were from subjects without asthma. However, the change in pERK1/2 expression in paediatric CD4^+^CD25^high^ T_regs_ cells was different compared with that observed in adults. ERK1/2 phosphorylation was observed at lower doses of H_2_O_2_ in CD4^+^CD25^high^ T_reg_ cells from paediatric subjects, and occurred more frequently, compared with adults. In addition, ERK1/2 phosphorylation was comparable between paediatric subjects with and without allergy (Figure 3b,c).

### 3.3. Age-Dependent Sensitivity of Jurkat Tet-On^®^ FOXP3 Cells to H_2_O_2_ Exposure In Vitro

**Figure 4:**
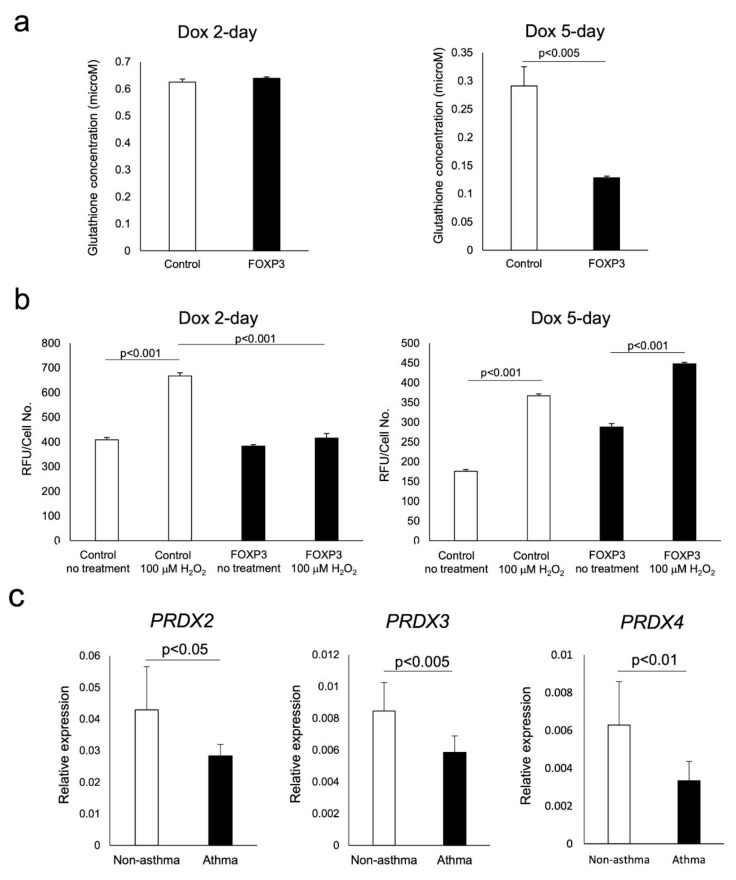
Assessment of the sensitivity of FOXP3^+^ cells to oxidative stress. The measurement of antioxidant levels and caspase activity in response to H_2_O_2_ were compared with those in control cells. Glutathione content in FOXP3 2-day and FOXP3 5-day cells were compared with control cells (**a**). Caspase activity in response in the abovementioned cells exposed to 100 μM H_2_O_2_ for 4 h were measured (**b**). mRNA expression of peroxiredoxins in CD4^+^CD25^+^CD127^−/low^ T cells were quantified by real-time PCR (**c**).

Jurkat cells inducibly overexpressing FOXP3 for 2 days (FOXP3 2-day cells) are similar to CD4^+^CD25^+^CD127^−/low^ T cells from subjects without asthma and FOXP3 5-day cells are similar to T_regs_ from asthmatic subjects. Sensitivity to oxidative stress was evaluated by H_2_O_2_-induced caspase activity, intracellular glutathione content, and microarray analysis. The glutathione content of FOXP3 2-day cells was similar to that measured in control cells; however, it was decreased in FOXP3 5-day cells (Figure 4a). H_2_O_2_-induced caspase activation in FOXP3 2-day cells was lesser than that observed in control cells treated with H_2_O_2_; however, caspase activation in FOXP3 5-day cells was restored to the level measured in control cells (Figure 4b). Microarray analysis revealed that peroxiredoxin (PRDX)-2, -3, and -4 were among the antioxidant genes that were increased distinctly in FOXP3 2-day cells, but not in control or FOXP3 5-day cells (Table 2). The results obtained by microarray were confirmed by measurement of the mRNA expression of these three genes in T_reg_ cells isolated from peripheral blood of adult subjects with or without asthma (Figure 4c).

## 4. Discussion

CD45RO, as a T_reg_ surface marker, is considered an indicator of T_reg_ maturity. CD4^+^FOXP3^low^CD45RA^+^ T cells are naive resting T_reg_ cells, whereas CD4^+^FOXP3^high^CD45RA^−^ T cells, like CD4^+^FOXP3^high^CD45RO^+^ T cells, constitute mature activated T_reg_ cells [23]. Both T_reg_ subtypes have a suppressive capacity in vitro, whereas phenotypically immature T_regs_ have the capacity to differentiate into phenotypically mature T_reg_ cells in vitro and in vivo. Previous reports indicated that phenotypically immature T_regs_ predominated in cord blood and in newborns [21,33]. Our data suggested that CD4^+^FOXP3^low^CD45RA^+^ T cells were dominant in peripheral blood before the age of 4 years, and that T_regs_ subsequently switched from an immature phenotype to a mature phenotype. Maternal antigen exposure is a crucial factor in phenotypic switching of CD45 T cell isoforms from pregnancy to infancy. Thereafter, phenotypic switching persists on further extrinsic antigen exposure that continues until adolescence. Similarly, T_reg_ cells have been suggested to undergo this type of conversion from immature to mature phenotypes in adults.

Several studies implicate low FOXP3 protein expression as a mechanism for abnormal T_reg_ function [6,7,8,9]. When we compared the characteristics of T_reg_ cells between paediatric and adult subjects with or without allergy, we observed that the phosphorylation of cellular signalling molecules ERK1/2 and p38 MAPK in T cells were increased by exposure to H_2_O_2_. Recently, T_reg_ cells were reported to have a high oxidative stress buffering capacity via increased secretion of thioredoxin-1 (TXN) [24]. When we examined T_reg_ cells with functional assays, we specifically observed that the T_reg_ cells from adults without asthma had attenuated pERK1/2 accumulation in response to H_2_O_2_ treatment, whereas the T_reg_ cells from adults with asthma were able to increase pERK1/2 under the same conditions. In addition, pERK1/2 was associated with robust Ca^2+^ influx in response to TCR stimulation in T_reg_ cells from adults with asthma. Previous reports demonstrated that the Ca^2+^ response of T_reg_ cells from children was correlated with the severity of asthma [11] and that of T_reg_ cells from adults was correlated with functional abnormalities of this cell type [12]. The Ca^2+^ response of adult T_reg_ cells can be divided into poor and robust response. Our findings indicated that robust pERK1/2 accumulation in T_reg_ cells from adults with asthma correlated with robust Ca^2+^ responses. Several studies suggested that ERK1/2 phosphorylation may occur following intracellular Ca^2+^ influx induced by exposure to H_2_O_2_ [27,34] and imply that robust Ca^2+^ response in T_reg_ cells may stimulate ERK1/2 phosphorylation. The increases in pERK1/2 and pp38 MAPK expression in response to oxidative stress were more robust in T_reg_ cells from paediatric subjects than those from adult subjects. These results imply that immature CD45RA^+^ T_reg_ cells were more sensitive to oxidative stress than CD45RO^+^ mature T_reg_ cells.

Our caspase activity studies showed that FOXP3 2-day cells were more tolerant to H_2_O_2_ than control cells, despite comparable glutathione content in both cell types, suggesting a distinct antioxidant response against oxidative stress. Microarray analysis revealed that peroxiredoxins, but not thioredoxin-1, were involved in tolerance to oxidative stress in our model system. In agreement with the results of microarray analysis of Tet-On^®^ Jurkat cells, mRNA expression of peroxiredoxin (PRDX)-2, -3, and -4 in T_reg_ cells from adults with asthma was decreased compared with T_reg_ cells from adults without asthma. Similarly, in Th17 cells, peroxiredoxin-2 was suggested to function as an antioxidant during chronic inflammatory processes [35]. We speculate that T_reg_ cells from adults with asthma were sensitized by oxidative stress with regard to inflammation; thus, the activation of ERK1/2 signalling was easily transduced. Chronic exposure to oxidative stress has been suggested to lead to death of T_reg_ cells in adults with asthma, compared with those in adults without asthma. The T_reg_ cells from paediatric subjects were sensitive to H_2_O_2_ exposure and Ca^2+^-responsive to TCR stimulation, irrespective of the presence of allergies in subjects [11], suggesting that T_reg_ cells from paediatric subjects were activated easily and were more susceptible to cell death. In summary, the phenotypic switching of T_reg_ cells were analysed based on the expression of CD45 isoforms during development. The high percentage of CD45RA^+^ T_reg_ cells in paediatric subjects may be reflected in their increased ERK1/2 signalling in response to H_2_O_2_ exposure. In contrast, T_regs_ from adults without asthma, most of which expressed CD45RO^+^, were tolerant to H_2_O_2_ exposure, whereas T_reg_ cells from adults with asthma were not. Increased tolerance of non-asthmatic adult T_reg_ cells may be attributable to intact upregulation of peroxiredoxins. Finally, assessment of MAPK phosphorylation in response to oxidative stress by flow cytometry is a simple and effective method for the evaluation of T_reg_ cell function in adults with asthma.

## 5. Conclusions

Developmental T_reg_ phenotypic switching occurs in early childhood, which may be a consequence of exposure to extrinsic antigens such as bacteria and viruses. The differential T_reg_ sensitivity to oxidative stress observed in children and adults was likely dependent on phenotypic CD45 isoform switching. Increased sensitivity of T_reg_ cells from adults with asthma to H_2_O_2_ resulted from a reduction of peroxiredoxin-2, -3, and -4 and increased pERK1/2 via impaired Ca^2+^ response in these cells.

## Figures and Tables

**Table 1:** Clinical characteristics of study subjects.

	Child		Adult	
	Allergy	Non-Allergy	Asthma	Non-Asthma
	(*n* = 38)	(*n* = 11)	(*n* = 10)	(*n* = 6)
Age	4.7 ± 3.5	4.0 ± 2.6	51.1 ± 20.1	29.3 ± 6.8
Gender (male/female)	28/10	6/5	6/4	4/2
Type				
Atopic	38		8	
Nonatopic	0		2	
Asthma severity				
Step 1	2			
Step 2	9		3	
Step 3	11		7	
Step 4	4			
Food allergy	15 (3) *^1^			
Atopic dermatitis	13 (7) *^2^			
Allergic rhinitis	2			
Dose of ICS				
CFC-BDP mg/day	183 (0–800)		492 (400–1600)	
Smoking				
Nonsmoker			7	6
Current smoker			3	0

^*1^, ^*2^: The number in parentheses indicates those further complicated by asthma.

**Table 2:** Microarray analysis.

		Control		FOXP3		Ratio
Symbol	Description	2-Day	5-Day	2-Day	5-Day	(FOXP3/Control 2-Day)
PXDN	Peroxidasin homolog Precursor (EC 1.11.1.7)(Vascular peroxidase 1)(Melanoma-associated antigen MG50)(p53-responsive gene 2 protein) [Source:UniProtKB/Swiss-Prot;Acc:Q92626]	69	50	230	184	3.33
TXNIP	Thioredoxin-interacting protein (Vitamin D3 up-regulated protein 1)(Thioredoxin-binding protein 2) [Source:UniProtKB/Swiss-Prot;Acc:Q9H3M7]	55	29	140	54	2.55
PRDX4	Peroxiredoxin-4 (EC 1.11.1.15)(Prx-IV)(Thioredoxin peroxidase AO372)(Thioredoxin- dependent peroxide reductase A0372)(Antioxidant enzyme AOE372)(AOE37-2) [Source:UniProtKB/Swiss-Prot;Acc:Q13162]	392	392	621	459	1.58
GPX4	Phospholipid hydroperoxide glutathione peroxidase, mitochondrial Precursor (PHGPx)(EC 1.11.1.12)(GPX-4) [Source:UniProtKB/Swiss-Prot;Acc:P36969]	1444	1151	2233	1832	1.55
SRXN1	Sulfiredoxin-1 (EC 1.8.98.2) [Source:UniProtKB/Swiss-Prot;Acc:Q9BYN0]	127	93	181	108	1.43
PRDX3	Thioredoxin-dependent peroxide reductase, mitochondrial Precursor (EC 1.11.1.15)(Peroxiredoxin-3)(PRX III)(Antioxidant protein 1)(AOP-1)(Protein MER5 homolog)(HBC189) [Source:UniProtKB/Swiss-Prot;Acc:P30048]	550	555	767	550	1.39
PRDX2	Peroxiredoxin-2 (EC 1.11.1.15)(Thioredoxin peroxidase 1)(Thioredoxin-dependent peroxide reductase 1)(Thiol-specific antioxidant protein)(TSA)(PRP)(Natural killer cell-enhancing factor B)(NKEF-B) [Source:UniProtKB/Swiss-Prot;Acc:P32119]	1972	1919	2378	1756	1.21
PRDX5	Peroxiredoxin-5, mitochondrial Precursor (EC 1.11.1.15)(Prx-V)(Peroxisomal antioxidant enzyme)(PLP)(Thioredoxin reductase)(Thioredoxin peroxidase PMP20)(Antioxidant enzyme B166)(AOEB166)(TPx type VI)(Liver tissue 2D-page spot 71B)(Alu corepressor 1) [Source:UniProtKB/Swiss-Prot;Acc:P30044]	1571	1910	1529	1978	0.97
TXN	Thioredoxin (Trx)(ATL-derivedfactor)(ADF)(Surface-associated sulphydryl protein)(SASP) [Source:UniProtKB/Swiss- Prot;Acc:P10599]	3510	3689	2338	1850	0.67
PRDX1	Peroxiredoxin-1 (EC 1.11.1.15)(Thioredoxin peroxidase 2)(Thioredoxin-dependent peroxide reductase 2)(Proliferation-associated gene protein)(PAG)(Natural killer cell-enhancing factor A)(NKEF-A) [Source:UniProtKB/Swiss- Prot;Acc:Q06830]	3861	3068	1303	1138	0.34

Shading shows increased gene expression in FOXP3 2-day cells compared with the others.

## Data Availability

Data are contained within the article or Supplementary Materials.

## Supplementary Materials

The following are available online at https://www.mdpi.com/article/10.3390/biomedicines9060616/s1: Figure S1: Microarray data.
