# Supplementary figures and images for: Surface Phenotype Changes and Increased Response to Oxidative Stress in CD4+CD25high T Cells
Source: Biomedicines. 2021 May 29;9(6):616. doi: 10.3390/biomedicines9060616 (PMC8229188; doi:10.3390/biomedicines9060616)

Figure S1. Microarray data

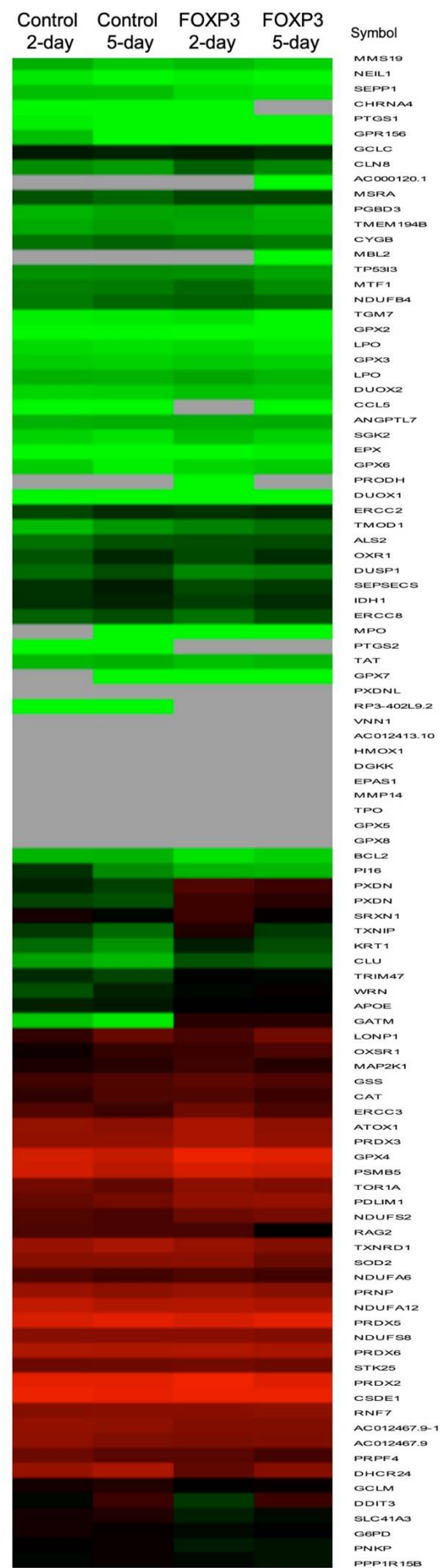

Supplement: Supplementary file 1 [file biomedicines-09-00616-s001.zip › biomedicines-1232297-supplementary.pdf]
